# Chimpanzee Autarky

**DOI:** 10.1371/journal.pone.0001518

**Published:** 2008-01-30

**Authors:** Sarah F. Brosnan, Mark F. Grady, Susan P. Lambeth, Steven J. Schapiro, Michael J. Beran

**Affiliations:** 1 Department of Anthropology, Emory University, Atlanta, Georgia, United States of America; 2 Michale E. Keeling Center for Comparative Medicine and Research, The University of Texas M. D. Anderson Cancer Center, Houston, Texas, United States of America; 3 Language Research Center, Georgia State University, Atlanta, Georgia, United States of America; 4 University of California, Los Angeles (UCLA) Center for Law and Economics, Los Angeles, California, United States of America; Claremont Graduate University, United States of America

## Abstract

**Background:**

Economists believe that barter is the ultimate cause of social wealth—and even much of our human culture—yet little is known about the evolution and development of such behavior. It is useful to examine the circumstances under which other species will or will not barter to more fully understand the phenomenon. Chimpanzees (*Pan troglodytes*) are an interesting test case as they are an intelligent species, closely related to humans, and known to participate in reciprocal interactions and token economies with humans, yet they have not spontaneously developed costly barter.

**Methodology/Principle Findings:**

Although chimpanzees do engage in noncostly barter, in which otherwise value-less tokens are exchanged for food, this lack of risk is not typical of human barter. Thus, we systematically examined barter in chimpanzees to ascertain under what circumstances chimpanzees will engage in costly barter of commodities, that is, trading food items for other food items with a human experimenter. We found that chimpanzees do barter, relinquishing lower value items to obtain higher value items (and not the reverse). However, they do not trade in all beneficial situations, maintaining possession of less preferred items when the relative gains they stand to make are small.

**Conclusions/Significance:**

Two potential explanations for this puzzling behavior are that chimpanzees lack ownership norms, and thus have limited opportunity to benefit from the gains of trade, and that chimpanzees' risk of defection is sufficiently high that large gains must be imminent to justify the risk. Understanding the conditions that support barter in chimpanzees may increase understanding of situations in which humans, too, do not maximize their gains.

## Introduction

Adam Smith famously argued that barter is the foundation of economic specialization, whereby one individual becomes a farmer, another becomes a hunter, and both get richer [1]. Although it is costly to each individual to give up something valuable (a bushel of wheat) in order to get back another valuable commodity (a pound of meat), in the end both individuals are better off for bartering because (economists believe) it is cheaper to produce just one commodity than all the different commodities one consumes [1]. Yet for all its benefits, barter is rarely seen outside of humans and we know little about its development.

In an autarkic society (that is, a society without barter), economic specialization cannot occur because each individual must self-produce all commodities consumed, or else rely on sporadic gifts. In order to fully understand the foundations of human economic behavior, it is critical to understand the development of basic behaviors, such as barter. Our closest living relative, the chimpanzee [2], shares many social and cognitive behaviors with humans, including reciprocal behavior [3]–[7], the use of token economies [8]–[13], and prototypic economic behaviors [10], [14], [15] (as do several other ape [16], [17] and monkey [18]–[22] species).

For instance, chimpanzees participate in reciprocal interactions that span many hours to weeks or months [3], [7]. Yet despite this, spontaneous exchange between chimpanzees is almost unknown. In laboratory settings, chimpanzees do exhibit token-based exchange behavior with humans [19–13, 23]. However, these interactions are noncostly for the chimpanzees, as the tokens have no use value if retained. Moreover, even in these token barter experiments, chimpanzees do not always trade to obtain the greatest level of reward possible (perhaps indicating that they do not understand the concept of ‘money’). Instead, chimpanzees (and capuchin monkeys) seem to focus on preferred rewards [10]. They show a strong preference for the token worth the greater reward (this association is taught via basic conditioning) and return it in all situations, whether or not the appropriate–higher value–food is available. Thus, they apparently focus on obtaining higher value rewards to the detriment of obtaining more rewards of all quality. Moreover, in situations in which tokens are given at the beginning of the trial and are not replenished, these primates return the tokens associated with higher value rewards first, thus ultimately limiting their acquisition of high-value foods (since they no longer possess the tokens necessary to acquire them).

In these studies, the tokens represented a sort of “money” for the chimpanzees. Given that chimpanzees do not use money in the wild, it is not surprising that chimpanzees do not use these tokens in the same way that humans do. They lack the extensive experience with such behavior that humans have. To more explicitly examine barter behavior in chimpanzees, we studied costly exchange behavior, in which subjects could barter food items with human experimenters to obtain other food items. This represents a far more natural situation for chimpanzees and thus provides a greater understanding of chimpanzee barter behavior.

Prior experience may play a dramatic role in cognition. For example, enculturation of chimpanzees leads to increased cognitive abilities, such as symbolic communication and enhanced imitative abilities [24], [25]. However, it is unknown whether such enculturation may facilitate exchange and barter behavior in chimpanzees. To examine the role of prior experience in barter, we tested subjects from two facilities with dramatically different rearing environments (see details below). Thus, if exposure to cognitive and linguistic training affected responses, we expect to see differences in barter behavior between these two populations.

## Results and Discussion

Ten subjects from the Michale E. Keeling Center for Comparative Medicine and Research of The University Texas M. D. Anderson Cancer Center, Bastrop, TX (Bastrop) had relatively little exposure to social and cognitive testing (although they did have daily contact with humans). These subjects were trained to exchange tokens shortly before the current study. Four additional subjects from the Language Research Center of Georgia State University, Atlanta, GA (LRC) had had extensive cognitive testing and (for three subjects) language training since infancy [25]. All were experienced with exchange with humans.

Not all subjects immediately generalized from exchanging tokens to exchanging foods. After initial token exchange training, Bastrop chimpanzees were given the opportunity to exchange air-popped popcorn, an undesirable food, for grapes, a desirable food, to verify that they extrapolated from exchanging tokens to exchanging food. Although subjects all expressed interest in the grapes, and offered to exchange many objects from their surroundings, they rarely offered the popcorn. Perhaps subjects hoped to receive the grape in return for something that was abundant in their environment, or perhaps they did not understand that the experimenter wanted the popcorn back in exchange for a grape. After additional food-exchange training, all subjects successfully exchanged carrot pieces for grapes.

Following this training, tests were begun. Foods used in the Bastrop experiments included carrots, apples, cucumbers, and grapes. To establish chimpanzees' preferences, required for the following experiments, we used a forced choice test that made them choose between two goods offered in pairs. Through this technique, we determined that the Bastrop subjects preferred grapes to carrots 90% of the time, grapes to cucumbers 83% of the time, and grapes to apples 79% of the time, for a descending preference order of grapes (favored), apples, cucumbers, and carrots.

Following exchange training, Bastrop subjects were given exchange sessions in which they were given 30 pieces of food wrapped in paper. These foods were completely under the chimpanzee's control and they could choose to exchange them on a one-to-one basis for preferred foods, displayed by the experimenter (see Methods). Subjects could also choose to consume these foods, or choose to consume some and exchange some in any order they preferred. The rate of consumption depended upon the desirability of the endowed food.

**Figure 1:**
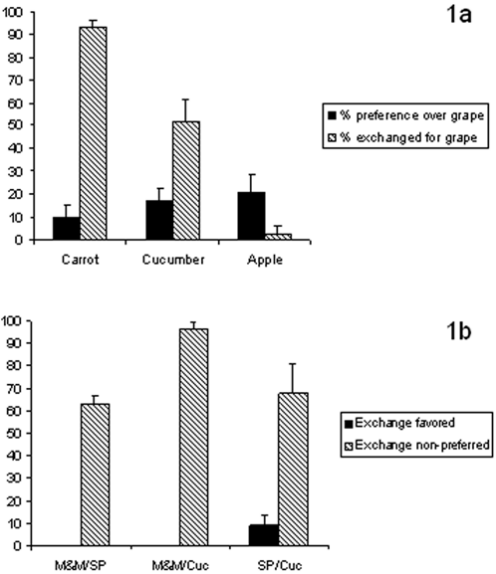
a. Chimpanzees were much less likely to exchange food commodities than their preferences would indicate when the endowed and available foods were close in value. Figure 1a indicates the percentage of times (±SE) chimpanzees at Bastrop chose to exchange a food for a grape (hatched bars) as compared to their preference for that food in comparison to grape (solid bars). Note that although grape was vastly preferred to all other foods, there was great variation in the willingness to give other foods up in return for a grape, with subjects exchanging virtually all carrots and virtually no apples. b. Indicates the percentage of times (±SE) chimpanzees at the Language Research Center chose to exchange one food for another. The x-axis indicates each food pairing; black bars indicate the percentage of times favored foods were exchanged for non-preferred foods and the hatched bars indicate the percentage of times non-preferred foods were exchanged for preferred foods. Favored and non-preferred were independently determined for each pair with respect to each other (favored foods are listed first for each category). As expected, subjects virtually never traded a favored food for a non-preferred food. However, subjects frequently chose to consume non-preferred foods rather than trade them for favored foods. Chimpanzees at Bastrop preferred higher value food items at least 80% over lower valued ones, and preferences were absolute for LRC chimpanzees (100% for M&Ms over both other foods, and 100% for sweet potato over cucumber). M&M = M & M brand chocolate candies, SP = uncooked sweet potato cubes, Cuc = raw cucumber pieces.

Initially, subjects were to receive two sessions each in which they were endowed with carrot, apple, and cucumber pieces (in that order). The chimpanzees virtually always bartered carrots for grapes (93% of the time; see Figure 1a), as one would predict based on their preferences, and there was no difference in the frequency of carrots traded for grapes compared to the frequency of choosing grapes over carrots in the preference test (χ^2 ^(N = 700) = 1.43, p>0.05). However, subjects almost never bartered apples for grapes (2%), exchanging less than expected based upon their preferences (χ^2 ^(N = 700) = 420.2, p<0.01). In case the barter of apple pieces for grapes did not reflect the foods' relative preferences in the preference test, a session was added in which subjects were endowed with 30 grapes and could exchange for apple pieces. Subjects never exchanged grapes for apple (0%), again exchanging less than anticipated based on preferences (χ^2 ^(N = 700) = 534.3, p<0.01).

The results of the cucumber exchanges were more variable, so we added 8 sessions (for a total of 10) to see if behavior stabilized. Overall, subjects exchanged 52% of their cucumber pieces for grapes, although this is still less than one would have anticipated based their preference for grapes over cucumbers (83% preference for grapes; χ^2 ^(N = 3,100) = 37.72, p<0.01).

**Figure 2:**
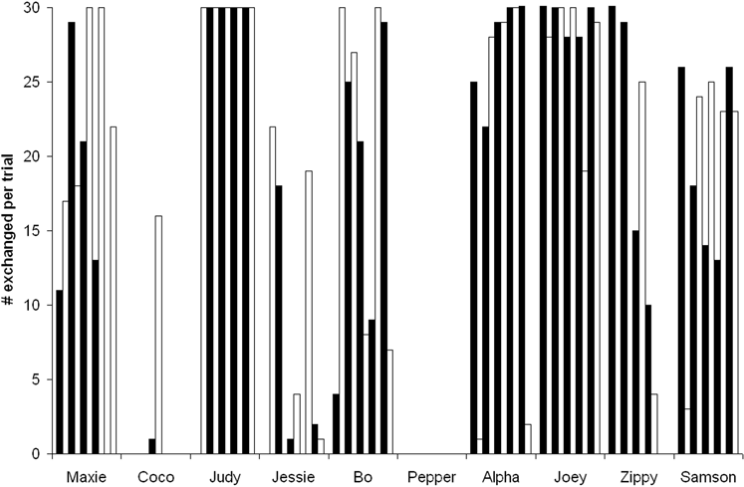
The number of exchanges by each subject at Bastrop in each of the 10 sessions in which they could exchange a piece of cucumber for a grape (30 exchanges were possible for each session). The number of exchanges was highly variable, both between subjects and between sessions within individuals. Sessions are presented in chronological order for each subject (indicated by their name), with black bars indicating odd sessions and white bars indicating even sessions.

Subjects' responses were highly variable. One individual never exchanged, while another exchanged every cucumber in 9 of 10 sessions (Figure 2). There was no correlation between the number of cucumbers exchanged for grapes and the strength of the preference for grape over cucumber (Spearman's rho correlation, rho(10) = 0.340, p = 0.337). However, individuals who began sessions with cucumber consumption rather than exchange were more likely to switch strategies than those who first exchanged for grapes (Spearman's rho correlation, rho(10) = 0.662, p = 0.037; 4 chimpanzees ate first, individual binomial tests p<0.05, 2 chimpanzees first exchanged, p<0.05, and 4 showed no preference). This indicates a link between delay of gratification or self control (which chimpanzees do exhibit [26]) and success in barter.

Overall, chimpanzees without extensive cognitive training do barter foods which are less valuable for those which are more valuable. However, this behavior is much more common for foods that differ greatly in value (e.g., grapes and carrots). Subjects had more difficulty making an expected exchange (based on their preferences) when foods were closer in value. In these cases the subjects tended instead to consume the less valuable food even when a more valuable food was available through barter.

The experiment at the LRC was similar, except that all exchanges of the three food types were offered in both directions. Thus, each chimpanzee was given six possible exchanges: high for medium, high for low, medium for high, medium for low, low for high, and low for medium. These chimpanzees were tested with three foods of a very clear preference order: M&M chocolate candies were the most preferred (preferred 100% of the time over both other options), then sweet potato pieces (uncooked; preferred to cucumbers 100% of the time), and then cucumber pieces (never preferred to the other options). Finally, due to previous experience, LRC chimpanzees did not require additional training to trade foods with human experimenters.

As with the Bastrop chimpanzees, the LRC chimpanzees kept virtually all of the endowment (i.e., did not exchange) when initially given a favored food (Figure 1b; 0% of M&Ms exchanged for sweet potatoes or cucumbers, 9% of sweet potatoes exchanged for cucumbers). Moreover, when there was a large difference between the less and more favored foods, and the chimpanzees were endowed with the less favored, they exchanged the majority of the time (96% of cucumbers exchanged for M&Ms; M&M-Cucumber, χ^2 ^(N = 240) = 441.6, p<0.01).

**Figure 3:**
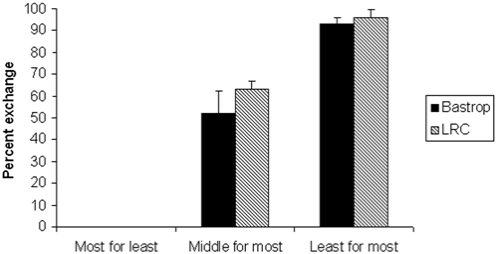
A comparison of the percent (±SE) of exchange behavior of the Bastrop and LRC subjects, showing very similar behavior between the enculturated (LRC) and non-enculturated (Bastrop) chimpanzees. As expected, chimpanzees from neither group ever exchanged the most preferred item (grape at Bastrop, M&M at LRC) for a less preferred item (apple at Bastrop, cucumber at LRC), and subjects typically exchanged the majority of their least preferred items (carrot at Bastrop, cucumber at LRC) for their most preferred items (grape at Bastrop, M&M at LRC). However, chimpanzees from neither group exchanged all of their middle value items (cucumber at Bastrop, sweet potato at LRC) for their most preferred items, even though the higher value items were highly preferred in other contexts.

However, for foods that were closer in value, the subjects showed less bartering of low value for high value foods than anticipated, based upon their barter of the same items in the other direction (high value for low value). For instance, when initially given sweet potatoes (medium value), chimpanzees exchanged only 63% for M&Ms (high value), yet no M&Ms were exchanged for sweet potato when M&Ms were the initial endowment (M&M-Sweet potato, χ^2 ^(N = 240) = 220.3, p<0.01). When the chimpanzees were initially given cucumbers (low value), they exchanged only 68% for sweet potatoes, whereas when endowed with sweet potato they exchanged only 9% (Sweet potato-Cucumber, χ^2 ^(N = 240) = 177.7, p<0.01). Thus, these chimpanzees were less significantly less likely to exchange a lower value food for a higher value food than they were likely to keep the higher value food when it was the endowment. In this, both LRC and Bastrop subjects behave similarly (Figure 3).

The fact that neither group of chimpanzees, with their radically different rearing histories, exchanged as much as predicted based on preferences when foods were close in value is strong evidence that chimpanzees find these exchanges problematic. However, two non-exclusive theories can explain this data.

First, the risk of defection discourages costly commodity barter. When a chimpanzee hands another individual a barter commodity, the second individual (let's say “the seller”) could defect and run away with both commodities. To the buyer, the expected cost of defection will be smaller the lower the value of the commodity that the buyer must hand over and the greater the reputation for cooperation possessed by the seller. The expected benefit from the barter to the buyer will be equal to the buyer's subjective difference in valuation between the commodity to be received and the sacrificed commodity (the “consumer's surplus”). This theory thus predicts that chimpanzee barter should take place more often when (1) the value of the sacrificed commodity is lower; (2) the seller's reputation for cooperation is higher; and (3) the “consumer's surplus” to the buyer is higher (as when a large difference in value exists between what the buyer has to give up relative to the promised commodity). Our experiments provide evidence in support of factors (1) and (3), as well as factor (2), because, as was clear to the chimpanzees, our human experimenters never defected.

A second, compatible, theory is that commodity barter probably cannot develop in the absence of ownership norms. Such norms allow individuals to lay down valuable commodities and store them for future barter or consumption; finding a barter partner while one is carrying a commodity would be a very rare occurrence. Chimpanzees do maintain possession norms (a kind of property norm) that protect commodities that they physically control, but an individual cannot specialize in production, or engage in large-scale barter, if the individual must hold its inventory in its hands. Property possession norms are less costly to enforce than property ownership norms because it is easier for an enforcer to witness and to correct a forcible dispossession than to decide which among competing claimants “owns” a commodity that one of them has set down. This property rights theory leads to the prediction that chimpanzee subjects should become more willing to barter when they possess increasingly secure hoards of commodities. Note that this theory is not counter to documented reciprocity in chimpanzees, because these interactions typically involve services, such as grooming or support, which do not require ownership norms, or even possession norms, to protect them. Thus, we expect exchange of services to emerge prior to exchange of commodities.

It is possible that both of the theories will prove to have explanatory value in future experiments on primate barter, and perhaps on human behavior as well, as humans, too, do not always maximize their gains in trade, especially when transaction costs are high. Further investigation of these theories could shed light on why chimpanzees do not engage in costly commodity barter and how this important form of cooperation became common among humans.

## Methods

Subjects included 10 adult chimpanzees drawn from a group living population housed at the Michale E. Keeling Center for Comparative Medicine and Research of The University of Texas M. D. Anderson Cancer Center, Bastrop, TX, USA and 4 adult chimpanzees from the Language Research Center at Georgia State University, Atlanta, GA. All studies were approved by the Institutional Animal Care and Use Committees of the centers which housed the chimpanzees. All subjects were housed in social groups with indoor/outdoor access and both food and material enrichment. No food deprivation was done, so subject motivation depended on the presence of favored treats. All subjects participated voluntarily, being called in from their social group and tested alone in the indoor area of their home enclosure. Sessions took approximately 10 minutes. Subjects received 1 session per day.

Chimpanzees were given a preference test for the food items involved in the study, consisting of a series of 10 forced-choice trials [27]. The side of each choice was alternated between trials. Chimpanzees always received the food they indicated.

Prior to this study, Bastrop subjects had been trained to barter inedible objects for food (LRC chimpanzees were already familiar with exchange). For a barter interaction, the experimenter's left hand was held outstretched, palm up, with the finger tips within a few inches of the caging. Upon returning the desired item, the chimpanzee was given a food reward.

In order that chimpanzees received all the endowed foods together during experimental tests, foods to be bartered were presented either loosely folded in a piece of butcher paper (Bastrop) or placed into a bowl (LRC). Prior to giving the endowment, the experimenter sat in front of the chimpanzee and counted out the exchange food in full view of the chimpanzee. The chimpanzee was then given one piece of each food to verify they knew what was available. Then the endowments were given to the subjects and barter began immediately.

For each barter interaction, the experimenter held up a piece of exchange food in the right hand and held out the left hand in a stereoypted “begging gesture,” hand outstretched with fingers near the mesh. The experimenter continued to talk to the chimpanzees to maintain a normal interaction, but no commands were given (e.g., “give”), nor were positive or negative words used (e.g., “good” or “no”). If the chimpanzee gave a piece of the endowment food to the experimenter, they received a piece of the exchange food in return. This was a one-to-one barter, so if they handed back 2 pieces, they got 2 pieces, and so forth. Then the experimenter immediately offered to barter again, with a new piece of the exchange food. The session ended when the chimpanzee had bartered or eaten the last piece of the endowed food, or when 3 minutes passed without an exchange or consumption of endowed food. The subject then got an unrelated food treat for participating. A second experimenter recorded the number of items eaten or bartered. Each chimpanzee always interacted with the same experimenter.

At Bastrop, subjects first received 2 sessions in which they were endowed with carrot (a thick slice cut in half). Following the “carrot sessions,” they received 2 “apple sessions,” which were identical to the above, except the endowment was apple pieces (1/16th of an apple). At the conclusion of the “apple sessions,” each chimpanzee received one “reverse” session, in which they were initially given grapes, which could be bartered for apple pieces. Finally, each chimpanzee received 10 “cucumber trials”. These were identical to above, except the endowment was cucumber pieces (each a slice cut in half). At the end of two sessions there was tremendous variability, hence the addition of eight more sessions.

At the LRC, subjects received 3 sessions of each condition in randomized order. The three conditions included cucumber (low value; a thick slice cut in half) for sweet potato (medium value; cubes approximately 2cm per side), cucumber for M&M (high value; a single candy), sweet potato for cucumber, sweet potato for M&M, M&M for cucumber, and M&M for sweet potato. Each session consisted of 20 trials.
